# *Aspergillus fumigatus* secretes a protease(s) that displays *in silico* binding affinity towards the SARS-CoV-2 spike protein and mediates SARS-CoV-2 pseudovirion entry into HEK-293T cells

**DOI:** 10.1186/s12985-024-02331-z

**Published:** 2024-03-06

**Authors:** Nozethu Mjokane, Eric O. Akintemi, Saheed Sabiu, Onele M. N. Gcilitshana, Jacobus Albertyn, Carolina H. Pohl, Olihile M. Sebolai

**Affiliations:** 1 Department of Microbiology and Biochemistry, University of the Free State, 205 Nelson Mandela Drive, Park West, 9301 Bloemfontein, South Africa; 2 Department of Biotechnology and Food Science, Faculty of Applied Science, Durban University of Technology, 4000 Durban, P.O. Box 1334, South Africa

**Keywords:** *Aspergillus (A) fumigatus*, *A. Fumigatus* alkaline protease 1, Haddock, HEK-293T cells, SARS-CoV-2 spike protein, Supernatant, Transduction

## Abstract

**Background:**

The novel coronavirus disease of 2019 (COVID-19) is an infectious disease caused by severe acute respiratory syndrome coronavirus-2 (SARS-CoV-2). Data from the COVID-19 clinical control case studies showed that this disease could also manifest in patients with underlying microbial infections such as aspergillosis. The current study aimed to determine if the *Aspergillus* (*A.*) *fumigatus* culture media (i.e., supernatant) possessed protease activity that was sufficient to activate the SARS-CoV-2 spike protein.

**Methods:**

The supernatant was first analysed for protease activity. Thereafter, it was assessed to determine if it possessed proteolytic activity to cleave a fluorogenic mimetic peptide of the SARS-CoV-2 spike protein that contained the S1/S2 site and a full-length spike protein contained in a SARS-CoV-2 pseudovirion. To complement this, a computer-based tool, HADDOCK, was used to predict if *A. fumigatus* alkaline protease 1 could bind to the SARS-CoV-2 spike protein.

**Results:**

We show that the supernatant possessed proteolytic activity, and analyses of the molecular docking parameters revealed that *A. fumigatus* alkaline protease 1 could bind to the spike protein. To confirm the *in silico* data, it was imperative to provide experimental evidence for enzymatic activity. Here, it was noted that the *A. fumigatus* supernatant cleaved the mimetic peptide as well as transduced the HEK-293T cells with SARS-CoV-2 pseudovirions.

**Conclusion:**

These results suggest that *A. fumigatus* secretes a protease(s) that activates the SARS-CoV-2 spike protein. Importantly, should these two infectious agents co-occur, there is the potential for *A. fumigatus* to activate the SARS-CoV-2 spike protein, thus aggravating COVID-19 development.

## Background

The novel coronavirus disease of 2019 (COVID-19) first emerged in China in 2019 [1, 2]. Since then, the disease has killed over 6.8 million people worldwide [3]. With time, it became apparent that COVID-19 could co-manifest with microbial infections [4, 5]. Clinical case-control studies of viral and fungal co-infections show that COVID-19 development places a sufficient burden on the host to allow for fungal infections to take hold. This is because the intense inflammatory response wherein inflammation-causing proteins flood the blood observed in COVID-19 patients– is reported to induce a pathological inflammatory disorder that allows for fungal infections to co-occur [6]. Additionally, the administration of immunomodulatory medicines such as corticosteroids in COVID-19 management– which are meant to suppress hyperinflammation, frequently leads to the development of COVID-19-associated mycotic infections [7]. One such infection is aspergillosis, which is caused by *Aspergillus fumigatus* [8]. Similar to the causative agent of COVID-19, i.e., SARS-CoV-2, inhalation of this fungus may result in a lung infection [9, 10]. Among COVID-19 patients, the fungus often presents between 4 and 11 days after intensive care unit admission, and the pooled COVID-19-associated pulmonary aspergillosis mortality is reported to be 56% [8]. Therefore, given the medical importance of this fungus– in part, being a serious COVID-19 complication, it was not surprising that it was recently included in the World Health Organization’s fungal priority pathogens list [11].

Our group has been concerned with a unique disease aspect that may develop when SARS-CoV-2 and some fungal species co-occur in the lung space, wherein fungal protease(s) could activate the viral spike glycoprotein (or spike protein). This is because fungi like *A. fumigatus* are reported to produce serine-based proteases essential for cellular growth and development, including virulence. In the context of virulence, these proteases help the fungus to disrupt the host structural barriers [12–14].

To invade a host cell, the viral particle uses the spike protein to mediate its entry into a cell [15]. As part of the entry mechanism, the spike protein reveals the fusion peptide that contains two regions known as S1 and S2 [16]. To release the fusion peptide, host proteases become critical in cleaving the spike protein at the S2 and the S1/S2 sites [17]. It is possible to foresee the S1/S2 site being catalysed by other proteases (including those of microbial origin), especially since enzymes that belong to the same family use common binding sites and have similar mechanistic features, which is defined by the nature of the nucleophilic amino acid in the active site [18]. Moreover, Fuller and co-workers [19] showed that the fungal serine protease, yeast kexin protease (present also in *A. fumigatus* [20]), is a fungal homologue of furin. Importantly, these proteases (furin and yeast kexin protease) share 50% gene sequence similarity [19, 21], which translates into significant functional homology [22]. Given the above, we sought to determine if *A. fumigatus* cultivation media (in the form of supernatant) may contain secreted serine protease(s) that could recognise the furin cleavage sites, i.e., the multibasic motifs of the preferred consensus sequence **R**-X-**R** or **R**-X-**K**↓, in the spike protein to activate it.

## Materials and methods

### Collection of *A. fumigatus* supernatant and detection of protease(s) in the supernatant

*A. fumigatus* strain was cultured on potato dextrose agar slants (PDA) agar (200 g/L potato infusion, 20 g/L dextrose, 20 g/L agar (Merck, South Africa)) and incubated for 24 h at 30 °C. Following this, a loopful of fungal colonies were picked and inoculated into a 250 mL conical flask that contained 100 mL of fresh, sterile YNB (6.7 g/L; Thermo Fisher Scientific, South Africa) broth that was supplemented with glucose (4%; w/v; Merck, South Africa). Next, the flask was then incubated at 30 °C for 32 h while agitated at 160 rpm on an orbital shaker (Lasec, South Africa). After 32 h, 25 mL of the culture media was then dispensed into a 50 mL centrifuge tube. The tube was centrifuged at 1000 × *g* for 5 min at 30 °C to pellet the cells and mobilise the *Aspergillus* protease(s) into the supernatant. To confirm the mobilisation of the protease(s) into the supernatant, it was tested with a Pierce™ Colorimetric Protease Assay Kit (Thermo Fisher Scientific) in accordance with the manufacturer’s protocol. The Kit detects the total protease activity in the tested sample.

### Molecular docking studies: protein acquisition, preparation, and molecular docking

The X-ray crystal structures of the SARS-CoV-2 spike glycoprotein (PDB ID: 6VXX) and *homo sapiens* furin protease (PDB ID: 5JXH) were obtained from the Research Collaboratory for Structural Bioinformatics (RCSB) Protein Data Bank (PDB) (https://www.rcsb.org) while the predicted structure of *A. fumigatus* alkaline protease 1 (PDB ID: AFUA_4G11800) was obtained from UniProt Protein Data Bank (https://www.uniprot.org). Table 1 shows the proteases used in this study.

**Table 1:** Furin and *A. fumigatus* alkaline protease 1 and their proposed function in the context of COVID-19 development

Protease	Type of protease	Source	Function(s) in source organism	Function in the context of SARS-CoV-2 infection
Furin protease (PDB ID: 5JXH)	Serine-based	*Homo sapiens*	Activates proprotein substrates [36].	Proteolytic cleavage of the viral S1/S2 site [25, 26, 35].
*A. fumigatus* alkaline protease 1 (PDB ID: AFUA_4G11800)	Serine-based	*Aspergillus fumigatus*	Help to disrupt the host structural barriers [14].	Suggested to act on the furin cleavage site within the S1/S2 site [35].

These structures were optimised by removing water molecules and non-standard naming protein residue connectivity [23]. Molecular docking of the structures: SARS-CoV-2 spike protein (target) and furin protease (ligand), or SARS-CoV-2 spike protein (target) and *A. fumigatus* alkaline protease 1 (ligand), were performed using the open-source computational tool known as the high ambiguity driven protein-protein docking (HADDOCK) (https://haddock.science.uu.nl) [24]. This tool was used to analyse the protein-protein interactions through the HADDOCK score, root-mean-square deviation (RMSD), Van der Waals energy, electrostatic energy, desolvation energy, restraints violation energy, and buried surface area.

### *A. fumigatus* supernatant and the proteolytic cleavage of the SARS-CoV-2 mimetic peptide

The fluorogenic assay was based on the protocols of Jaimes and co-workers, however, with minor modifications [25, 26]. Briefly, the prepared reaction conditions were reported to activate the hydrolysis of most substrates by furin [27] and, by extension, other serine-based proteases. Moreover, the used substrate contained an amino acid sequence (underlined, SPRRAR↓S) that is highly susceptible to furin hydrolysis, as any mutation to the sequence would impair furin hydrolysis.

A reaction mixture for the synthesized fluorogenic mimetic peptide was carried out in a 100 µL buffer solution (pH 7.5) composed of (1) 100 mM Hepes (Merck, South Africa), (2) 0.5% Triton X-100 (Merck, South Africa), (3) 1 mM CaCl_2_ (Merck, South Africa), and (4) 1 mM 2-mercaptoethanol (Merck, South Africa). Furin was diluted to 10 U/mL, and 0.5 µL was added to the reaction mixture. In a separate experiment, 0.5 µL of the *A. fumigatus* supernatant was added. Reactions were performed at 30 °C, and a fluorometer measured fluorescence emission every minute for 45 min. Fluorescence intensity was tracked over this time interval using the wavelength settings, i.e., excitation (λ355 nm) and emission (λ405 nm). Six independent experiments were carried out, and the means Vmax was calculated.

### *A. fumigatus* supernatant and the transduction of HEK-293T cells by SARS-CoV-2 pseudovirion

#### Cultivation of HEK-293T cells

The HEK-293T cells were maintained in DMEM medium that was supplemented with 10% (v/v) foetal bovine serum (Biochrom, Germany), 20 U/mL penicillin (Sigma-Aldrich), and 20 mg/mL streptomycin (Sigma-Aldrich). For each biological repeat, the cells were grown at 37 °C and 5% CO_2_ until they reached 80% confluence, and their viability was determined using the trypan blue stain (Sigma-Adrich, USA). Next, the cells were standardised to reach a final cell concentration of 1 × 10^4^ cells/mL in 10 mL of fresh DMEM (Sigma-Aldrich, USA). A 50 µL suspension of the HEK-293T cells was seeded into wells of a sterile, white disposable 96-well flat-bottom microtitre plate (Greiner Bio-One, Germany) and left overnight in a 5% CO_2_ incubator at 37 °C.

#### SARS-CoV-2 pseudovirion entry assay

The protocol from BPS Bioscience for transducing HEK-293T cells using the SARS-CoV- 2 spike pseudotyped Lentivirus (transfecting vector with all the accessory proteins for viral entry and infectivity - but devoid of the genomic material) was used (BPS Bioscience, United States). This pseudovirion also contains the firefly luciferase gene. Following overnight incubation, the spent media was aspirated. A total of 5 µL of the pseudovirion and 44.5 µL of fresh DMEM media were added to the same wells. To initiate pseudovirion entry, 0.5 µL of the recombinant furin (New England Biolabs, United States) or 0.5 µL of the *A. fumigatus* supernatant was added. The plate was incubated overnight at 37 °C with 5% CO_2_. The next day, the spent media was aspirated, and 50 µL of fresh DMEM media was added. The plate was incubated for 60 h at 37 °C with 5% CO_2_. A bald pseudovirion that lacks the SARS-CoV-2 spike protein (BPS Bioscience) was included as a control.

#### The use of the one-step™ luciferase assay to measure infectivity

The assay was performed according to the manufacturer’s protocol (BPS Bioscience). The kit consisted of two components, A (reagent buffer) and B (reagent substrate). In brief, the luciferase reagent buffer (component A) was thawed at room temperature. The components (A + B) were mixed to make a working solution at a 1:100 ratio. The luciferase assay working solution component (A + B) was added directly to the culture medium using an equal volume to the volume of the culture medium, which was 50 µL. The cells were incubated for 30 min while gently rocking at room temperature. After that, luminescence was measured using a Fluoroskan Ascent FL, which converts logarithmic signals to relative luminescence units (RLUs). The luminometer was programmed to perform a 10-sec measurement delay followed by a 20-sec measurement read for luciferase activity. As the DMEM was supplemented with foetal bovine serum, which may contain proteases, it was included as a negative control. In the end, the background signal of this negative control was used for normalisation.

### Statistical analysis

Where applicable, for each study, three independent experiments were performed. No technical repeats were included for each independent experiment. GraphPad Prism 8.0 (GraphPad Software, Inc., United States) was used to calculate the mean values, standard error mean (SEM), and the statistical significance of the data was considered at *p* < 0.05.

## Results

### The *A. fumigatus* supernatant contains protease(s) that theoretically activate the SARS-CoV-2 spike protein

Analyses of the Pierce protease assay data confirmed that the tested supernatant contained protease activity when compared to absorbance readings obtained against the standard protease provided in the Kit (data not shown). And by necessary implication, we hypothesised that the *A. fumigatus* alkaline protease 1 is present in the tested supernatant. Therefore, to determine if the observed supernatant activity could translate into activation of the SARS-CoV-2 spike protein, the docking results were considered. Here, the *in silico* comparative analysis data of the docking parameters of SARS-CoV-2 spike protein with furin protease or *A. fumigatus* alkaline protease 1 is detailed in Table 2.

**Table 2:** The intermolecular binding energies of the docked furin-SARS-CoV-2 spike protein complex and *A. fumigatus* alkaline protease 1-SARS-CoV-2 spike protein. HADDOCK.

Protease	Z-score	RMSD (Å)	HADDOCK score	Van der Waals energy (kcal/mol)	Electrostatic energy (kcal/mol)	Desolvation energy (kcal/mol)	Restraints violation energy (kcal/mol)	Buried surface area (Å^2^)
Furin protease (PDB ID: 5JXH)	-1.0	28.4 ± 0.0	-79.6 ± 0.1	-50.9 ± 0.1	-128.1 ± 7.8	-3.6 ± 1.5	4.9 ± 1.0	1796.7 ± 18.9
*A. fumigatus* Alkaline protease 1 (PDB ID: AFUA_4G11800)	-2.7 ± 0.0	0.0 ± 0.0	-156.2 ± 0.0	-77.9 ± 0.0	-308.6 ± 0.0	-16.5 ± 0.0	0.0 ± 0.0	3471.7 ± 0.0

The clustering default for parameters for furin protease-SARS-CoV-2 protein complex had a cluster minimum size of one while for *A. fumigatus* alkaline protease-1-SARS-CoV-2 had a cluster minimum size of two

We recorded a HADDOCK score of -79.6 ± 0.1 when furin protease-SARS-CoV-2 spike protein was docked. In contrast, the docking of *A. fumigatus* alkaline protease 1-SARS-CoV-2 spike protein gave a significantly negative score of -156.2 ± 0.0 (*p* < 0.05) compared to the furin protease. The average RMSD value and Z-score of furin protease in complex with SARS-CoV-2 spike protein were 28.4 ± 0.0 Å and − 1.0, and that of *A. fumigatus* alkaline protease 1 were 0.0 ± 0.0 Å and − 2.7 ± 0.0 (Table 2). The Van der Waals energy and electrostatic energy that contribute to the binding strength for each complex were calculated. For the furin protease-SARS-CoV-2 spike protein complex, the Van der Waals and electrostatic energy values were − 50.9 ± 0.1 kcal/mol and − 128.1 ± 7.8 kcal/mol, respectively. Compared to these, *A. fumigatus* alkaline protease 1-SARS-CoV-2 spike protein complex had scores of -77.9 ± 0.0 kcal/mol (Van der Waals energy) and − 308.6 ± 0.0 kcal/mol (electrostatic energy).

The buried surface area, i.e., the amount of protein surface not in contact with water upon complexation, data was calculated and analysed. Here, it was noted that the *A. fumigatus* alkaline protease 1 had a score of 3471.7 ± 0.0 Å^2^ while that of the furin protease (1796.7 ± 18.9 Å^2^). The furin protease’s desolvation energy (-3.6 ± 1.5 kcal/mol) and restraint violation energy (4.9 ± 1.0 kcal/mol) were observed to be far less than the buried surface area score (1796.7 ± 18.9 Å^2^). Similar readings were obtained for the *A. fumigatus* alkaline protease 1. This protease had a score desolvation energy of -16.5 ± 0.0 kcal/mol and restraint violation energy of 0.0. ± 0.0 kcal/mol (Table 2).

**Fig. 1:**
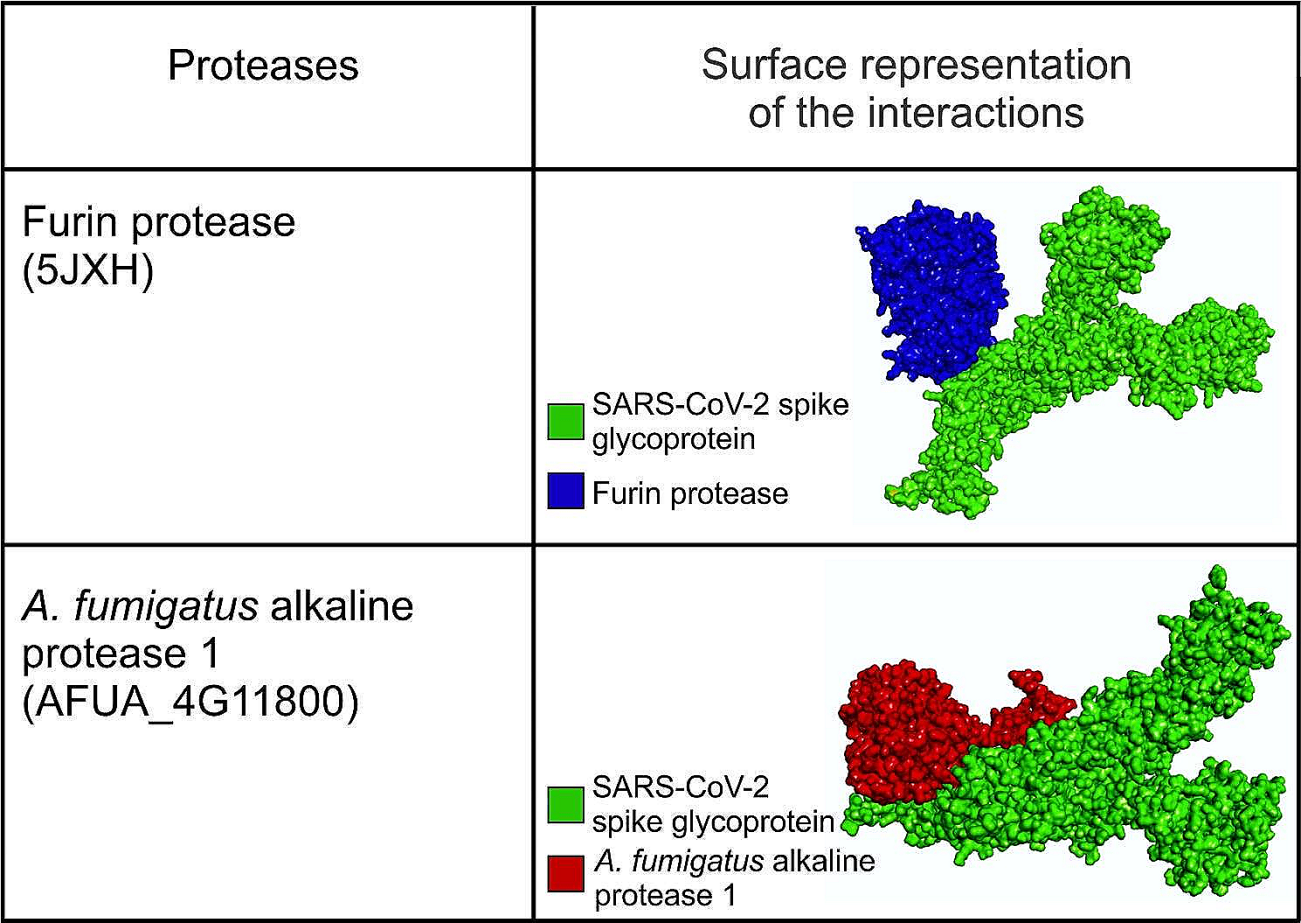
The surface representation of the docked furin protease-SARS-CoV-2 spike protein complex and *A. fumigatus alkaline* protease 1-SARS-CoV-2 spike protein complexes. Discovery Studios Visualizer (v21.1.0.20298) was used for the interactive visualisation and analysis of the molecular structures

**Fig. 2:**
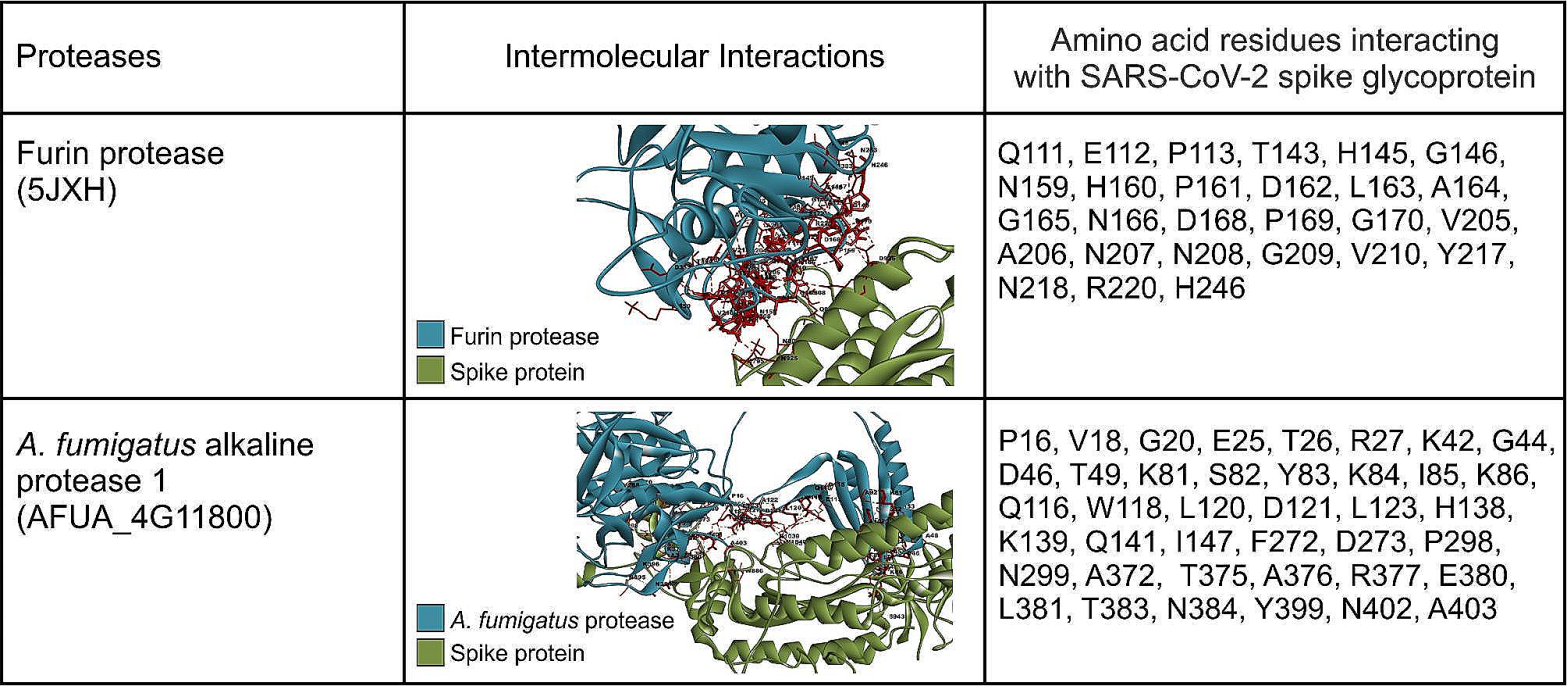
The intermolecular interactions of the docked furin protease-SARS-CoV-2 spike protein complex *and A. fumigatus* alkaline protease 1-SARS-CoV-2 spike protein showing the amino acid residues of each protease that interacted with the spike protein. Discovery Studios Visualizer (v21.1.0.20298) was used for the interactive visualisation and analysis of the molecular structures

**Fig. 3:**
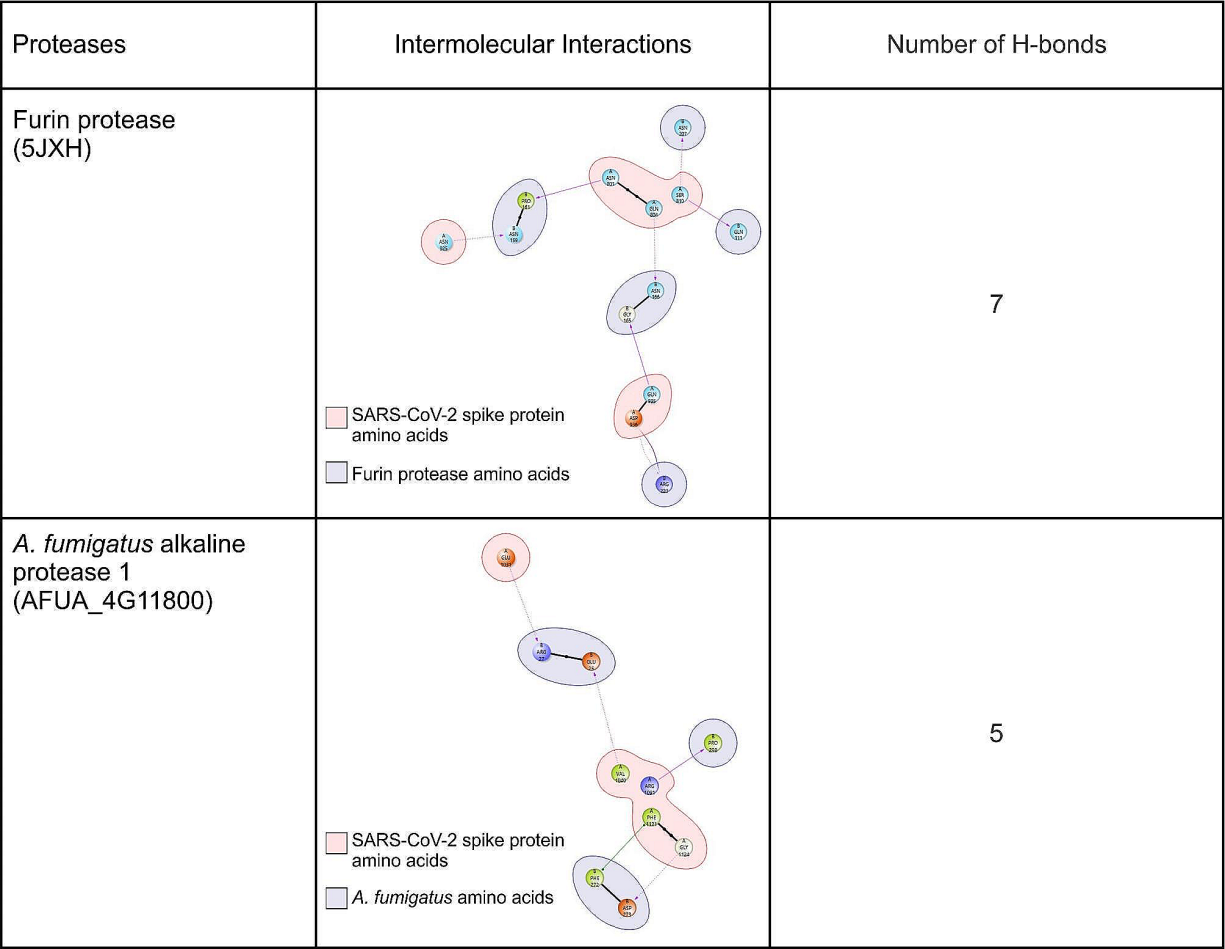
The intermolecular interaction of the docked furin protease-SARS-CoV-2 spike protein complex and *A. fumigatus* alkaline protease 1-SARS-CoV-2 spike protein showing the H-bonds for each protease and the SARS-CoV-2 spike protein. Discovery Studios Visualizer (v21.1.0.20298) was used for the interactive visualisation and analysis of the molecular structures

In Fig. 1, we provide information related to the surface representation of the docked complexes, i.e., furin protease-SARS-CoV-2 spike protein and *A. fumigatus* alkaline protease 1-SARS-CoV-2 spike protein– at their respective optimal orientation. We further summarise the characteristic amino acid residues for each protease that were poised to interact with the spike protein cleavage site in the docked complex structure, in Fig. 2. The furin protease interacted with Q111, E112, P113, T143, H145, G146, N159, H160, P161, D162, L163, A164, G165, N166, D168, P169, G170, V205, A206, N207, N208, G209, V210, Y217, N218, R220, and H246 at the binding pocket of the spike protein. In contrast, *A. fumigatus* alkaline protease 1 interacted with P16, V18, G20, E25, T26, R27, K42, G44, D46, T49, K81, S82, Y83, K84, I85, K86, Q116, W118, L120, D121, L123, H138, K139, Q141, I147, F272, D273, P298, N299, A372, T375, A376, R377, E380, L381, T383, N384, Y399, N402, and A403. In addition, the furin protease-SARS-CoV-2 spike protein complex was linked by 7 hydrogen bonds, while the *A. fumigatus* alkaline protease 1-SARS-CoV-2 spike protein complex had 5 bonds (Fig. 3).

### Enzymatic evidence of *A. fumigatus* supernatant activating the SARS-CoV-2 spike protein

**Fig. 4:**
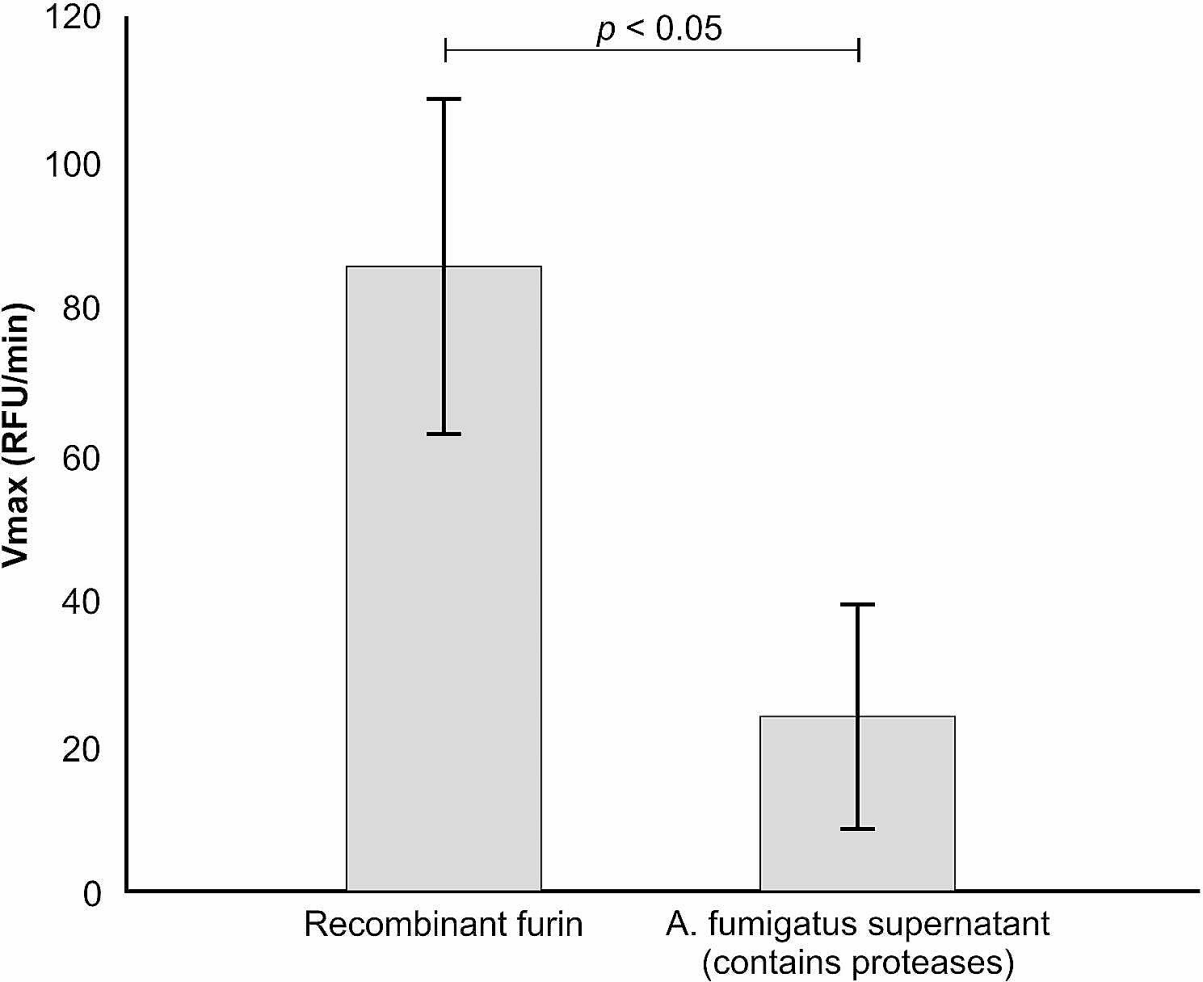
A measure of the proteolytic reaction following the cleavage of a fluorescent peptide viz. viz. TNSPRRARSVA (SARS-CoV-2; S1/S2 site), by the furin protease or *A. fumigatus* supernatant. Vmax = maximum velocity, RFU = Relative Fluorescence Unit

The fluorogenic assay data showing the biochemical efficiency of furin protease or *A. fumigatus* supernatant cleaving a mimetic peptide that contains an amino acid sequence (underlined, SPRRAR↓S) is summarised in Fig. 4. This unique amino-acid sequence is present at the interface between the S1 and S2 site and serves as a cleavage site for the human furin protease. We determined that the *A. fumigatus* supernatant mediated the proteolytic cleavage of the fluorogenic peptide, however, not in a manner that was comparable to the efficiency of recombinant furin (*p* < 0.05).

**Fig. 5:**
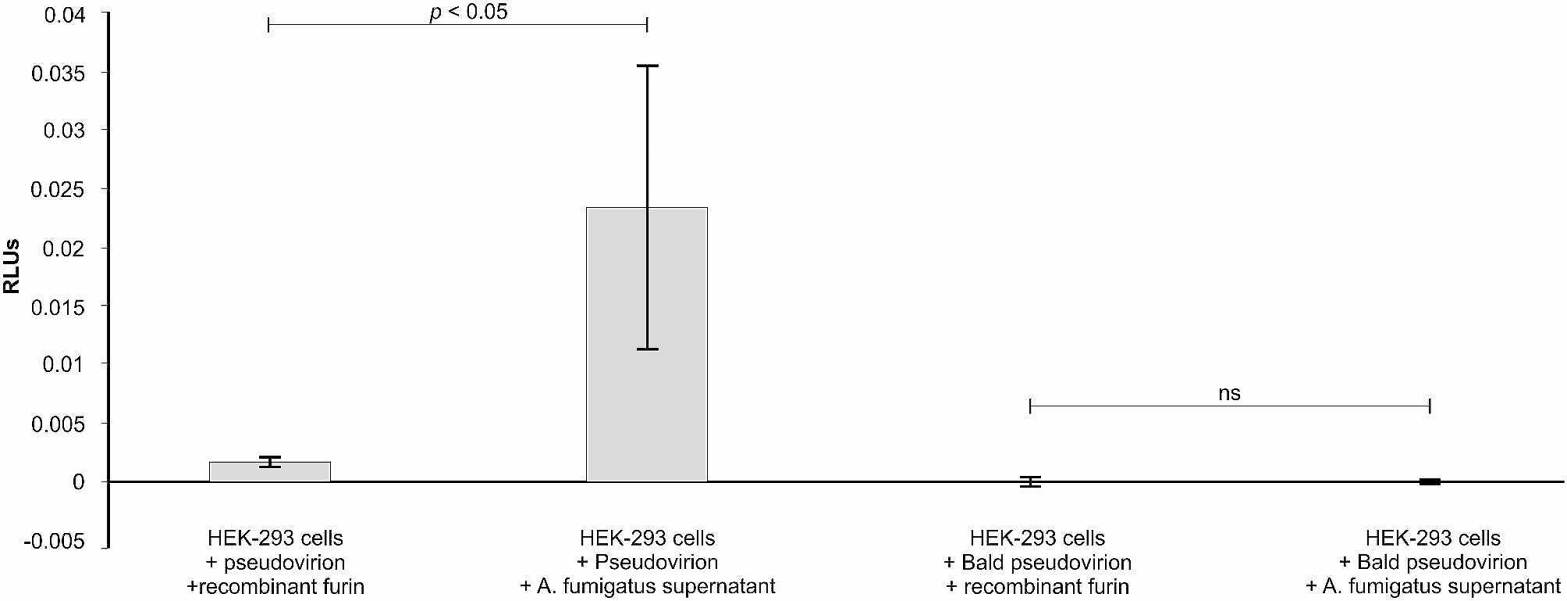
The SARS-CoV-2 spike pseudovirion infectivity in HEK-293T cells in the presence of recombinant furin or *A. fumigatus* supernatant. The cells were infected with pseudovirion or bald pseudovirion, and their luciferase activity was measured. Error bars indicate the standard error measurements of three biological replicates. The SARS-CoV-2 spike pseudovirion contains the luciferase gene and the spike protein. The bald SARS-CoV-2 spike pseudovirion contains the luciferase gene but lacks the spike protein. ns = not significant; RLUs = Relative Luminiscence Units

A major drawback of using mimetic peptides is that they might not be cleaved in a similar manner as a purified full-length SARS-CoV-2 spike protein - due to differences in conformation; thus, these peptides may not resemble the original folding of the full-length protein as argued by Jaimes and co-workers [22]. Therefore, it was crucial that we validate our findings by using a full-length spike protein present in pseudovirions in a transduction study. To this end, we summarised our infectivity results in Fig. 5. It was noted that the biochemical efficiency of *A. fumigatus* supernatant to transduce HEK-293T cells with SARS-CoV-2 pseudovirions was greater than that of the recombinant furin (*p* < 0.05). As expected, zero and close to zero relative luminescence units were obtained when the bald pseudovirion was in the presence of either recombinant furin or *A. fumigatus* supernatant. These obtained results confirm that *A. fumigatus* supernatant contains a protease (serine-based) that is able to cleave the fluorogenic peptide and transduce the HEK-293T cells.

## Discussion

While the WHO announced the end of the COVID-19 global pandemic in 2023 [28], SARS-CoV-2 remains a threat that continues to cause infections in susceptible hosts. And more importantly, the virus could co-manifest with fungal infectious agents. Unfortunately, aspergillosis has emerged as a COVID-19 complication that is often associated with high mortality [29]. With this paper, we sought to determine the potential of *A. fumigatus*’ protease(s) in activating the SARS-CoV-2 spike protein– thus, contributing to COVID-19 development.

We ran comparative analyses using furin protease as a reference serine-based protease because it is secreted by human cells, and it is reported to participate in the cleavage of the SARS-CoV-2 spike protein at the S1/S2 site [30, 31]. Our *in silico* data revealed that the *A. fumigatus* alkaline protease 1 could fit at the binding domain of the SARS-CoV-2 spike protein based on its negative HADDOCK docking score. Interestingly, its docking score was more negative than that of the furin protease. With HADDOCK, the more the negative score, the better the orientation and high binding affinity of an entity at the binding domain of a protein [32]. The buried surface area of *A. fumigatus* alkaline protease 1 (3471.7 Å^2^) was greater than that of the furin protease (1796.7 Å^2^). This suggests that the binding affinity between *A. fumigatus* alkaline protease 1-SARS-CoV-2 spike protein was stronger, with a larger binding surface area than that of furin protease-SARS-CoV-2 spike protein. The observed amino acid differences highlight that the two proteases had different orientation preferences in the binding site. It was noted that protein-protein interactions for both complexes, viz. the furin protease-SARS-CoV-2 spike protein and the *A. fumigatus* alkaline protease 1-SARS-CoV-2 spike protein, were dominated by electrostatic energy, which is essential for the catalytic triad to perform the covalent catalysis [14]. Additionally, the furin protease-SARS-CoV-2 spike protein complex had more hydrogen bonds, showing a strong interaction between the protein and the ligand [33].

Taken together, the docking data suggested that *A. fumigatus* alkaline protease 1-SARS-CoV-2 spike protein formed the best compact macromolecular complex compared to furin protease-SARS-CoV-2 spike protein as it was a better ligand to interact with the SARS-CoV-2 spike protein than furin protease. Thus, in turn, it could theoretically activate the SARS-CoV-2 spike protein.

To test the above theory, it was prudent to show if the *A. fumigatus* supernatant possessed enzymatic activity. Based on the Pierce™ colorimetric protease assay results, it was concluded that the supernatant displayed protease activity. Thus, it was not surprising to observe that the proteases contained in the supernatant could cleave the mimetic peptide used in this study. The designed mimetic peptide is only susceptible to hydrolysis by a serine-based protease. This is because the early characterisation of the SARS-CoV-2 spike protein revealed a unique site, i.e., constituted by a sequence of four amino acids (underlined, SPRRAR↓S) at the interface between the S1/S2 site, that serves as a potential cleavage site for furin protease [25, 26]. And that, any mutation to this amino acid sequence would impair furin hydrolysis. From the docking data, we established that furin protease and *A. fumigatus* alkaline protease 1 belong to the same family, thus would use common binding sites and have similar mechanistic features, i.e., defined by the nature of the nucleophilic amino acid in the active site [18, 34]. Therefore, it is reasonable to foresee *A. fumigatus* alkaline protease 1 (serine-based) catalysing the SARS-CoV-2 spike protein similarly to furin protease. The idea of a fungal protease activating a viral substrate has been shown before. In their work, Mjokane and co-workers showed that the fungus *Cryptococcus neoformans* secretes protease(s) that can activate the spike protein of SARS-CoV-2 [35]. As a mimetic peptide may not fold in a similar manner as a full-length peptide, it was imperative to show if the *A. fumigatus* supernatant contained a protease(s) that could transduce a laboratory cell line following the activation of the SARS-CoV-2 spike protein. From the analyses, it was noted that, like the furin protease, *A. fumigatus* alkaline protease 1 could cleave the S1/S2 site to create an unstable receptor-binding domain that, in turn, led to endocytosis of the pseudovirion by HEK-293T cells.

In conclusion, we demonstrated the potential of fungal protease(s) in activating a viral protein. It, therefore, becomes important to demonstrate if other viral proteins, e.g. the influenza haemagglutinin, that participate in viral entry could also be activated by fungal proteases. Concerning the current study, it is also critical to isolate fungal protease(s) and determine if they can activate a full-length peptide leading to host invasion. Should the latter hold, this will have major implications for COVID-19 patients. This is because it is conceivable that the virus would have access to a broad pool of mammalian and microbial furin-like proteases that could be perverted to ensure host invasion. As a control strategy, it would be critical to determine if COVID-19 patients have a fungal infection, which ought to be treated. Moreover, the administration of an anti-protease drug (protease inhibitor compound) could help regulate virus-fungal interaction, including virus-host interaction. However, the latter should be to the exclusion of side effects.

## Data Availability

All data generated or analysed in this study are included in this published article.
